# The Role of Long-Range Non-Specific Electrostatic Interactions in Inhibiting the Pre-Fusion Proteolytic Processing of the SARS-CoV-2 S Glycoprotein by Heparin

**DOI:** 10.3390/biom15060778

**Published:** 2025-05-28

**Authors:** Yi Du, Yang Yang, Son N. Nguyen, Igor A. Kaltashov

**Affiliations:** Department of Chemistry, University of Massachusetts-Amherst, Amherst, MA 01003, USA; yidu@umass.edu (Y.D.); yyang@umass.edu (Y.Y.); sonngoc.nguyen@umassmed.edu (S.N.N.)

**Keywords:** coronavirus, furin, polyanion, glycosaminoglycans, non-covalent complex, multi-valence, avidity, native mass spectrometry, limited charge reduction

## Abstract

The proteolytic processing of the SARS-CoV-2 spike glycoprotein by host cell membrane-associated proteases is a key step in both the entry of the invading virus into the cell and the release of the newly generated viral particles from the infected cell. Because of the critical importance of this step for the viral infectivity cycle, it has been a target of extensive efforts aimed at identifying highly specific protease inhibitors as potential antiviral agents. An alternative strategy to disrupt the pre-fusioviden processing of the SARS-CoV-2 S glycoprotein aims to protect the substrate rather than directly inhibit the proteases. In this work, we focused on furin, a serine protease located primarily in the Golgi apparatus, but also present on the cell membrane. Its cleavage site within the S glycoprotein is located within the stalk region of the latter and comprises an arginine-rich segment (SPRRARS), which fits the definition of the Cardin–Weintraub glycosaminoglycan recognition motif. Native mass spectrometry (MS) measurements confirmed the binding of a hexadecameric peptide representing the loop region at the S1/S2 interface and incorporating the furin cleavage site (FCS) to heparin fragments of various lengths, as well as unfractionated heparin (UFH), although at the physiological ionic strength, only UFH remains tightly bound to the FCS. The direct LC/MS monitoring of FCS digestion with furin revealed a significant impact of both heparin fragments and UFH on the proteolysis kinetics, although only the latter had IC50 values that could be considered physiologically relevant (0.6 ± 0.1 mg/mL). The results of this work highlight the importance of the long-range and relatively non-specific electrostatic interactions in modulating physiological and pathological processes and emphasize the multi-faceted role played by heparin in managing coronavirus infections.

## 1. Introduction

SARS-CoV-2 is no longer considered a global health threat, but the virus continues to circulate worldwide, with the number of new cases reported weekly to the World Health Organization consistently exceeding 10,000 in the past six months (and the weekly numbers of COVID-19-related deaths staying consistently above 500), despite many countries no longer collecting these data. While the fatality rates have decreased dramatically since the peak of the pandemic, the long-term effects of COVID-19 are now being increasingly recognized as a significant concern [1]. The continuous evolution of SARS-CoV-2 diminishes the efficacy of both prevention and treatment [2], placing a premium on finding therapeutic options that are less sensitive to the virus mutations that give rise to the cornucopia of the variants of concern (VOCs). Heparin is a highly sulfated linear polysaccharide from the glycosaminoglycan (GAG) family that was shown two decades ago to reduce the infectivity of other coronaviridæ (e.g., pre-COVID SARS-CoV [3]), and it emerged early in the COVID-19 pandemic as a potential treatment [4,5,6,7,8]. The antiviral properties of heparin were initially attributed to its ability to associate with the receptor-binding domain of the viral spike glycoprotein (S RBD). Specifically, it was postulated that heparin blocks the docking of S RBD to its host cell surface receptor (angiotensin-converting enzyme 2, ACE2) due to an overlap of the heparin- and ACE2-binding interfaces on the S RBD surface [9]. This conjecture was confirmed in a study that used a combination of native mass spectrometry (MS) and molecular modeling to characterize the interaction of heparin derivatives with S RBD and their inhibition of the S RBD/ACE2 association [10,11].

The interaction between polyanionic heparin and S RBD is driven by long-range electrostatic forces and depends on the presence of an extensive patch of a positive charge on the surface of the latter, which is critical for its interaction with the low-pI ACE2 [10]. However, S RBD is not the only segment of the S glycoprotein that harbors a heparin-binding interface. Indeed, Linhardt and co-workers [12] noted that the polybasic furin cleavage site within the S1/S2 junction (an important structural determinant of SARS-CoV-2 virulence, which is not found in closely related coronaviruses [13]) fits the definition of the Cardin–Weintraub GAG recognition motif [14] and, therefore, lends itself as an alternative site for heparin binding. This finding has been interpreted in terms of the possible involvement of the host cell’s surface GAGs in the initial anchoring of the viral particle within the extracellular matrix of the host cell and facilitating its subsequent entry into the cell [12]. However, another possible—yet still overlooked—implication of heparin binding at the furin cleavage site is its likely interference with the pre-fusion proteolytic processing of the S glycoprotein.

The important role played by furin and other host proteases vis-à-vis the pre-fusion processing of the S glycoprotein [15] put this enzyme in the crosshairs of medicinal chemistry research early in the pandemic [16]. While the efforts to identify highly specific inhibitors of furin continue unabated [17], the selective protection of its cleavage site within the S glycoprotein (as opposed to protease inhibition) may offer an alternative antiviral strategy that would not compromise the ability of this proprotein convertase to execute a plethora of its cellular household tasks [18]. Heparin (and perhaps other polyanionic GAGs) may lend itself as an attractive candidate in this respect, as its interaction with the polybasic furin cleavage site should be governed primarily by relatively non-specific electrostatic interactions. Such interactions should be largely insensitive to mutations within the furin cleavage site unless they result in abolishing (or significantly reducing) its polybasic character. The latter, however, will likely render furin ineffective vis-à-vis being able to recognize and proteolytically process such segments, thereby impacting the virulence of the affected strains (in fact, there is a multitude of evidence that both the loss of the furin cleavage site and the elimination of the basic residues within this segment result in a dramatic attenuation of infectivity [19,20]). Furthermore, the majority of SARS-CoV-2 VOCs that have arisen over the course of the pandemic harbor mutations within the furin cleavage site and feature an increase in the number of basic residues within this segment (e.g., P681H in Alpha, P681R in Delta and N679K/P681H in Omicron) [15]. While all these changes enhance the cleavage by furin (and facilitate cell entry, likely contributing to an increased transmissibility) [21], they should also result in a higher affinity to polyanions (such as heparin), thereby amplifying the protective properties of the latter.

In this work, we used a combination of native MS, enzymatic reaction kinetics measurements with LC/MS and molecular modeling to study the interaction of both heparin fragments and full-length unfractionated heparin (UFH) with the model peptide YQTQTNSPRRAR(↓)SVAS, which represents residues 674–689 of the wild-type S glycoprotein and maps onto the furin cleavage site. This model peptide (termed the FCS) associates with both heparin derivatives and unfractionated heparin (UFH), with the latter being able to accommodate multiple FCS molecules within a single polyanion chain at the physiological ionic strength. This association endows the FCS with notable protection against furin. The presence of three furin cleavage sites within the S glycoprotein trimer is likely to multiply this effect, since a heparin chain as short as 30 monosaccharides (15 glucosamine/uronic acid pairs) can bridge two distinct furin cleavage sites within the trimer, and the avidity arising due to the di- and (possibly) tri-dentate interactions will further amplify the protective properties of heparin. These findings emphasize the importance of relatively non-specific electrostatic interactions in physiology and highlight the previously neglected therapeutic modality of heparin in antiviral applications.

## 2. Materials and Methods

### 2.1. Chemicals and Biologicals

UFH and the heparin fixed-length fragments dp6 and dp20 were purchased from Galen Laboratories (North Haven, CT, USA). Fondaparinux and H_2_O^18^ were purchased from Millipore-Sigma (St. Louis, MO, USA). The dissolution of heparinoids in deionized water resulted in nearly neutral solutions (pH range: 6.5 to 8.0). No additional pH adjustments were made prior to the MS measurements, as the pKa values of both carboxylic and sulfate groups fall far outside of this range, and furin retains its optimal enzymatic activity within a broader pH range (5.5–8.5) [22]. The synthetic peptide representing the furin cleavage site (FCS) of the original isolate sequence of SARS-CoV-2 S (UniProt: P0DTC2) was purchased from Biomatic (Cambridge, ON, Canada). The recombinant form of the ectodomain of human furin was acquired from BioLegend (San Diego, CA, USA). All biologicals were used without further purification. All solvents and buffers used in this work were of analytical grade or higher.

### 2.2. Mass Spectrometry (MS)

The native MS measurements of FCS interactions with fondaparinux, dp6 and dp20 were carried out using a SolariX 7 (Bruker Daltonics, Billerica, MA, USA) FT ICR mass spectrometer. The following source settings were used to minimize the loss of sulfate from heparin in the gas phase: dry gas temperature, 200 °C; collision voltage, 1 V; DC extract bias, −0.4 V; skimmer 1 potential, −20 V. The FCS association with UFH was monitored with a Synapt G2si (Waters Corp., Milford, MA, USA) hybrid quadrupole/time-of-flight mass spectrometer equipped with a nanospray source. The following ion source parameters were used to maintain noncovalent complexes in the gas phase: capillary voltage, 1.5 kV; sampling cone voltage, 80 V; source offset, 80 V; trap CE, 4 V; trap DC bias, 3 V; and transfer CE, 0 V. This instrument was also used for limited charge reduction measurements. Ion selection for limited charge reduction was achieved by setting the appropriate quadrupole selection parameters (LM resolution set at 4.5). Limited charge reduction was initiated by allowing the *m*/*z*-selected multiply charged ions to interact with 1,3-dicyanobenzene anions for 0.6 ms after setting the trap wave height as 0.3 V and optimizing the discharge current. FCS digestion by furin was carried out at room temperature using the following concentrations: furin, 0.2 ng/μL; FCS, 0.1 μg/μL; heparinoids, 1 μg/μL). Proteolysis kinetics measurements were carried out using an Orbitrap Fusion (Thermo-Fisher, Waltham, MA, USA) LC/MS system. Chromatographic separation was performed using a Dionex UltiMate 3000 HPLC system (Dionex Corporation, Sunnyvale, CA, USA) equipped with an Agilent ZORBAX RR Extend-C18 analytical column (2.1 mm × 50 mm, 3.5 µm; Agilent, Santa Clara, CA, USA). The column was maintained at 25 °C. The mobile phase consisted of (A) 0.1% formic acid (FA) and 30 mM ammonium acetate (NH_4_Ac) in water, and (B) 0.1% FA and 30 mM NH_4_Ac in 85% acetonitrile. The gradient elution program was as follows: 3% B from 0 to 1 min, 10% B at 2 min, 15% B at 5 min, 90% B at 6 min, and 3% B from 7 to 11 min. The flow rate was set at 0.2 mL/min. The MS analysis was conducted in the positive ion mode, with the mass spectra acquired over an *m*/*z* range of 300–1500 at a resolution of 60,000 (*m*/*z* 200), using a spray voltage of 3500 V and a maximum injection time of 50 ms and an AGC target of 4 × 10^5^. Data-dependent acquisition (DDA) was performed with a 3 s cycle time, where precursor ions were selected for fragmentation via higher-energy collisional dissociation (HCD) with a collision energy of 30%. Fragment ions were recorded at a resolution of 15,000, with an isolation width of 1.6 *m*/*z* units, a maximum fill time of 22 ms, and an AGC target of 5 × 10^4^. The presence of polyanions in the FCS solution was found to result in a significant deterioration of the quality of RPLC-MS chromatograms when using common mobile phases (based on 0.1% formic acid). The chromatographic process fidelity was restored upon the addition of a moderate amount of an ESI-compatible weak electrolyte (30 mM ammonium acetate) to both mobile phases.

### 2.3. Molecular Modeling

All molecular modeling work was carried out using a Maestro (Schrödinger LLC, New York, NY, USA) modeling suite, release 2019-4. The initial dp6 model for the FCS/dp6 docking studies was prepared using the PDB 3IRJ structure as a template (by extracting a hexameric segment from the reducing end). At known sulfation sites, the sulfate group occupancy was randomized to yield 7–9 sulfate groups per chain. To maintain the expected ionization state of the sulfate groups during docking, the input partial charges were preserved throughout the ligand preparation and docking process. The fully sulfated dp20 model was extracted from the PDB 3IRJ structure by deleting a tetrasaccharide segment from the nonreducing end of the heparin dp24 chain. The model peptide was constructed from the known sequence YQTQTNSPRRARSVAS. Docking and molecular dynamics (MD) simulations were set up using the Glide and Desmond applications with an OPLS3e force field, respectively [23,24]. All the initial models were energy-minimized until the maximum change in the distance between the atoms dropped below 0.1 Å prior to the docking and simulation studies. The receptor grid was generated using Glide, centered on the peptide with the grid encompassing the entire peptide. Standard grid generation settings were used, with the van der Waals radius scaling factor set to 1.0 and a partial charge cutoff of 0.25. Molecular docking was performed using Glide in standard precision mode, with post-docking minimization enabled. The best docking poses were selected based on the GlideScore values and the binding energies were estimated using the MM-GBSA method implemented in Prime. All visualizations were prepared using Maestro. See the Supporting Information for the detailed molecular simulation conditions.

## 3. Results

### 3.1. Molecular Modeling of the Interaction Between Heparin Fragments and the Furin Cleavage Site of the SARS-CoV-2 S Glycoprotein

**Figure 1:**
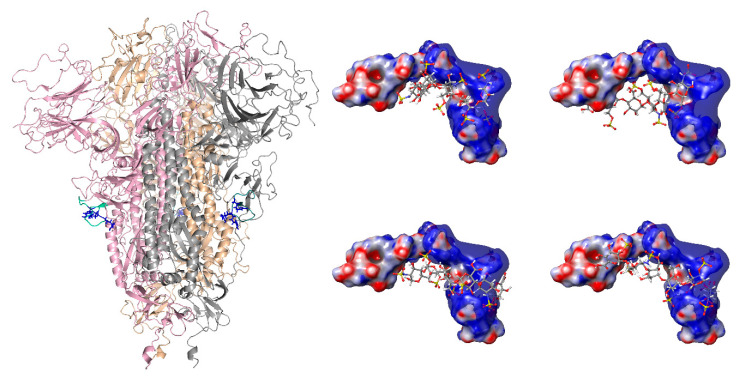
(**Left**): Structure of the trimeric form of the S glycoprotein (PDB 6VXX), with each monomeric unit colored individually (pink, wheat, and gray), showing the furin cleavage sites in green (the side chains of the three arginine residues in each monomeric unit are colored in blue). (**Right**): The top docking poses of the FCS with dp6, showing four different binding conformations (see Table S1 for MMGBSA results).

The furin cleavage site of the SARS-CoV-2 S glycoprotein is located within a polypeptide segment that has an anomalous degree of conformational motility, as predicted by multiple algorithms comprising the PONDR (Predictor of Natural Disordered Regions) engine [25], nor is this segment visible in the crystal structures of the protein [26,27,28]. Therefore, a polypeptide with an amino acid sequence corresponding to this unstructured region can be used as a realistic model of this part of the S glycoprotein. We chose a sixteen-residue-long peptide, YQTQTNSPRRARSVAS (residues 674-689 of the original SARS-CoV-2 S glycoprotein), as a model of the protein segment containing the furin cleavage site. Molecular dynamics simulations demonstrated that this peptide (which will be referred to as the FCS) readily associates with a heparin oligomer as short as five saccharides in length (the synthetic heparin mimetic fondaparinux [29]). In addition, an MD simulation of the fully sulphated heparin hexamer dp6 species and two FCS molecules showed that dp6 can readily bridge two FCSs (Figure S1). While fondaparinux and the fully sulfated dp6 used in the MD simulation are chemically defined molecules, endogenous polyanions, such as heparin, exhibit significant heterogeneity at different levels (e.g., the distribution of chain lengths, as well as the distribution of sulfate groups within each chain). The molecular dynamics studies highlight the importance of electrostatic interactions as determinants of the association of the polybasic FCS and heparin oligomers of fixed lengths varying in both the extent of sulfation and its distribution across the polysaccharide (Figure 1 and Figure S1). Importantly, the molecular modeling studies yielded an ensemble of structures of the peptide/heparin complexes, consistent with the notion that the relatively non-specific electrostatic interactions allow for a significant conformational freedom within such complexes as opposed to favoring a single well-defined conformation [30].

### 3.2. Native MS of FCS Interactions with Short-Chain GAGs

**Figure 2:**
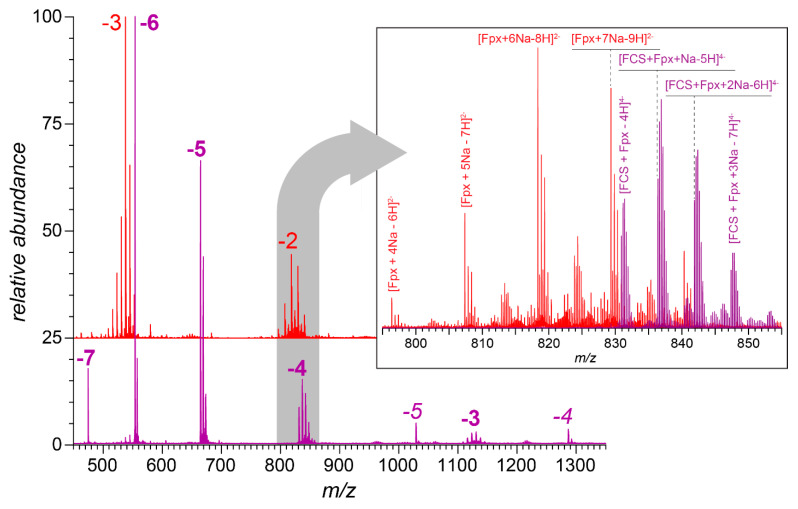
Negative ion mass spectra of fondaparinux (29 μM in deionized water, red trace) and the fondaparinux/FCS mixture (equimolar amounts in deionized water, purple trace). The charge states of fondaparinux anions are indicated with red-colored numerals, and the multiplicity of peaks in each charge state was due to the varying degree of sodium adducts (e.g., the most abundant peak in the −3 cluster corresponds to the [M-8H+5Na]^3−^ ions, and the second most abundant peak in the same cluster corresponds to the [M-9H+6Na]^3−^ ions). The charge states for the fondaparinux/FCS complexes are shown in purple (bold type for 1:1 complexes and italics for 1:2 complexes). The inset shows zoomed views of the mass spectral regions in *m*/*z* 795–855.

Consistent with the molecular modeling predictions, the native MS revealed the strong propensity of the FCS to associate with short heparin oligomers, as is exemplified by the formation of non-covalent complexes between the FCS and fondaparinux. The mass spectrum of a nearly equimolar mixture of fondaparinux and the FCS in deionized water featured an abundant ionic signal corresponding to the 1:1 (with a minor contribution from 1:2) polysaccharide/peptide complex (Figure 2). The signal of free fondaparinux ions was clearly absent from the spectrum, indicating its near-complete consumption (the presence of the 1:2 polysaccharide/peptide complex ions in the mass spectrum suggested that there was a slight molar excess of the peptide in the mixture).

**Figure 3:**
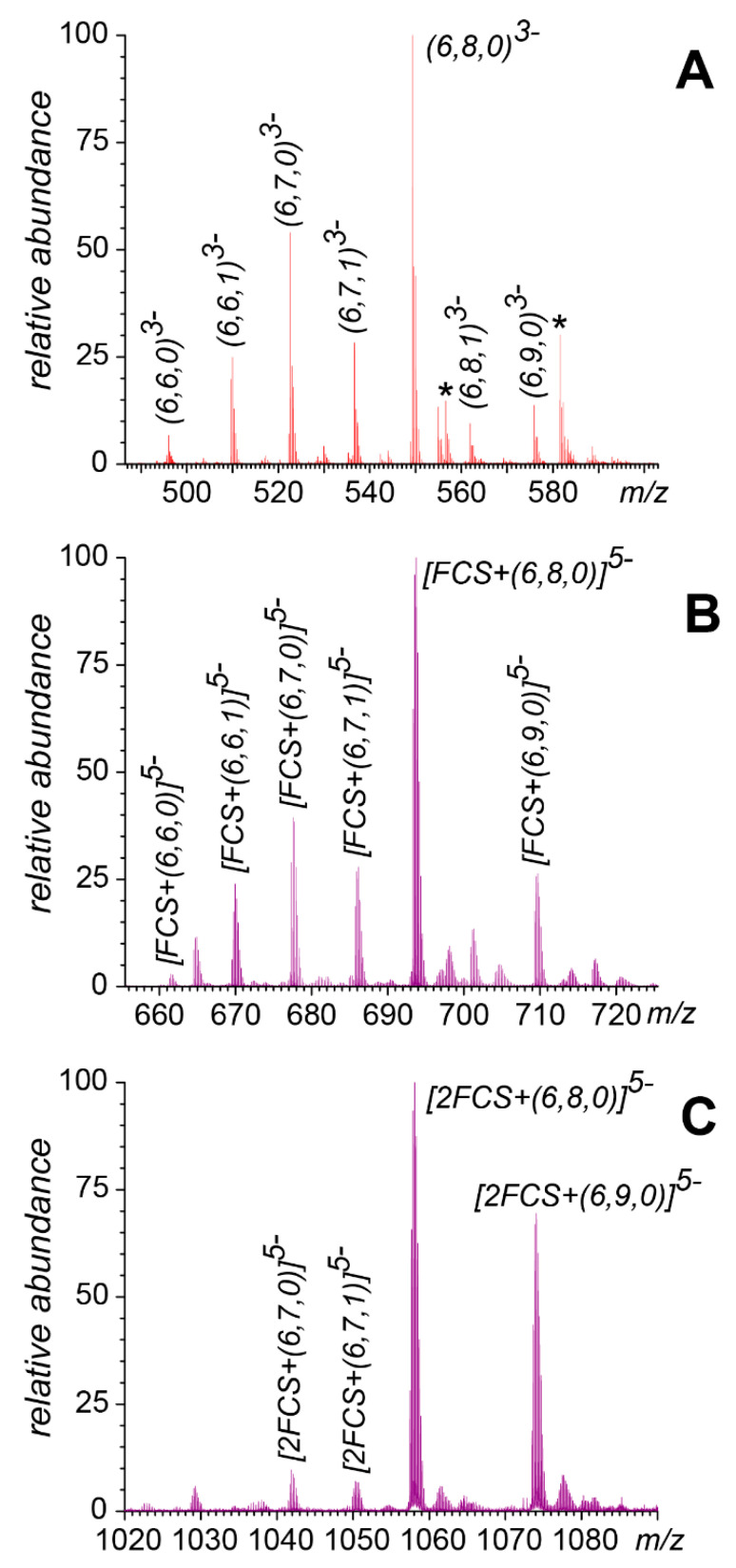
Ionic intensity distributions for heparin hexamers with different degrees of sulfation and acetylation in mass spectra of dp6 alone (**A**) and a nearly equimolar mixture of dp6 and FCS (**B**,**C**). The displayed spectral regions correspond to free dp6 at charge state −3 (**A**), a 1:1 dp6/FCS complex at charge state −5 (**B**), and a 1:2 dp6/FCS complex at charge state −5 (**C**). The asterisks (*) indicate Na+ adducts. The labeling of individual species follows Henriksen’s nomenclature [32] (the three numbers in parentheses identify the number of saccharide units in the chain, the number of sulfate groups, and the number of acetyl groups, respectively). See Figure S2 in Supporting Information for the full-range mass spectra.

Unlike fondaparinux, heparin fixed-length fragments (such as heparin hexamers, dp6) are produced from the natural product (UFH) via partial enzymatic depolymerization. Therefore, dp6 molecules feature a notable degree of heterogeneity due to the presence of species with varying degrees of sulfation and acetylation [31]. This is evident in the mass spectrum of dp6 (Figure 3A), which showed a distribution of ionic intensities for seven such species with distinct masses (the peaks are labeled following Henriksen’s nomenclature [32], i.e., the numbers in parentheses indicate the chain length, the number of sulfate groups, and the number of acetyl groups within the chain). Mixing dp6 with a nearly equimolar amount of the FCS in deionized water resulted in the complete elimination of the dp6 signal from the mass spectrum, while giving rise to abundant 1:1 dp6/FCS complexes; the 1:2 complexes were also observed (Figure 3C). The ionic intensity distribution of the 1:1 dp6/FCS complexes as a function of the number of sulfate groups within the heparin hexamer closely mirrored the dp6 anion intensity distribution in the absence of the peptide in solution (compare panels A and B in Figure 3). However, the 1:2 dp6/FCS complexes (Figure 3C) displayed a markedly different pattern, with only the highly sulfated dp6 species being capable of bridging two polybasic peptides (the full-range mass spectra are shown in Figure S2). This indicates the critical importance of electrostatic interactions in the formation of such complexes: bringing two polycationic peptides (each containing three arginine residues) into close proximity by a heparin hexamer requires at least seven sulfate groups, but can be accomplished most effectively by hexamers incorporating the maximum possible amount (nine) of sulfate groups. The native MS also revealed a robust FCS/polyanion association for longer fixed-length heparin oligomers, with the dp20 species capable of accommodating up to eight peptides (see the Supporting Information for more detail). However, all these interactions were highly sensitive to the presence of other electrolytes in the solution and were nearly eliminated even before the ionic strength reached a physiological level (Figures S3–S5).

### 3.3. Native MS of FCS Interactions with UFH

**Figure 4:**
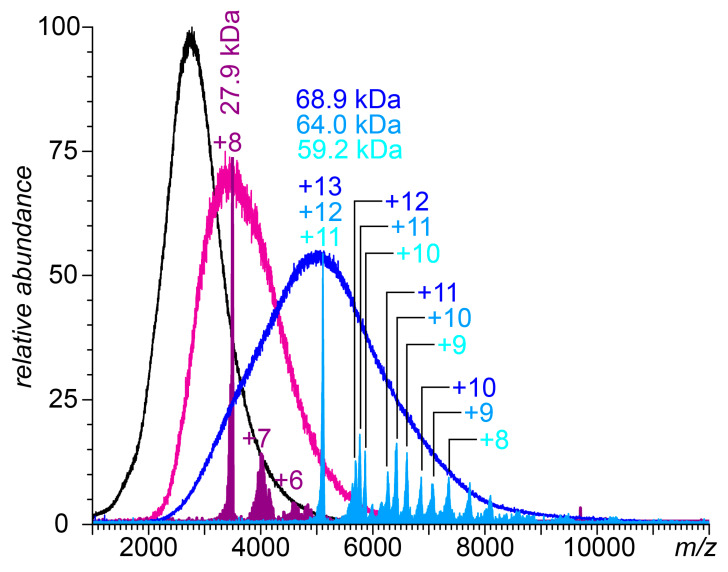
Native mass spectra of 0.43 mg/mL aqueous solutions of UFH in 150 mM ammonium acetate and 0.53 and 2.64 mg/mL FCS (magenta and blue traces, respectively). The color-filled curves represent the results of limited charge reduction measurements performed on ionic populations selected within *m*/*z* windows 3475–3525 (purple) and 5050–5150 (teal). The peaks are labeled with the corresponding charge states (as deduced from the charge ladders).

The extreme structural heterogeneity exhibited by intact UFH gives rise to a continuum ionic signal distribution across a wide *m*/*z* range from which no distinct charge states can be discerned and, as a result, no meaningful mass information can be obtained in conventional MS measurements. This problem can be dealt with by using the so-called limited charge reduction, a technique that relies on generating well-resolved charge ladders off ionic populations selected within narrow *m*/*z* windows by exposing them to either electrons or electron-donating radicals in the gas phase [33]. The use of a limited charge reduction allows meaningful information to be extracted from the native MS data on systems as complex as UFH associations with antithrombin [34] and heparin-containing immune complexes [35]. The UFH preparation used in this work has been previously characterized using a combination of native MS and limited charge reduction, revealing a wide mass range (8–42 kDa), with the most abundant signal corresponding to 11–14 kDa (corresponding to the polysaccharide chain lengths of 40–50 monosaccharide units) [35]. Addition of the FCS to the UFH solution at a significant molar excess (10- to 50-fold) resulted in a notable shift in the ionic signal to the higher *m*/*z* region (Figure 4). The masses of the most abundant ions determined by the limited charge reduction (28 kDa and 59–69 kDa for 10- and 50-fold peptide molar excesses, respectively) indicate that a single heparin chain can associate with a significant number of FCS peptides (ca. ten at the 10-fold molar excess and 25–30 at the 50-fold molar excess, assuming that the highest signal in each case corresponds to the most abundant heparin chains). Unlike the shorter heparin oligomers, the UFH/FCS interactions persisted at higher salt levels (the data presented in Figure 4 were acquired at the physiological ionic strength).

### 3.4. Attenuation of FCS Proteolysis with Furin by Fondaparinux, Fixed-Length Heparin Fragments, and UFH

**Figure 5:**
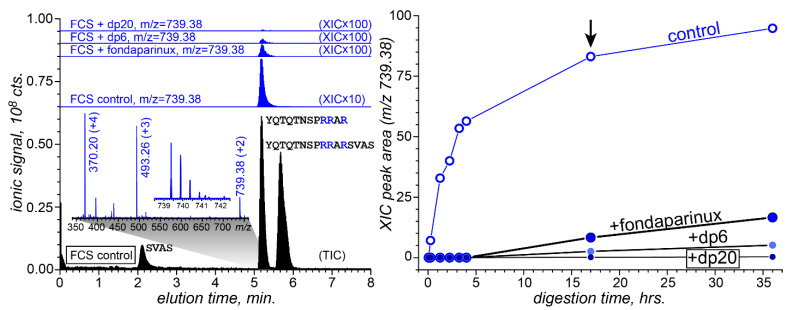
(**Left**): The total ion chromatogram (TIC) of FCS (10 μg) digestion products following its 17 min exposure to furin (20 ng) in a low-ionic-strength solution at 25 °C (black-filled curves) and extracted ion chromatograms (XICs, blue) of ions representing the fragment peptide YQTQTNSPRRAR (charge state: +2) from the digestion mixture in the absence and presence of heparinoids (as stated on individual chromatograms). (**Right**): The abundance of the proteolytic fragment as a function of the digestion time. The black arrow indicates the time point for which the LC/MS data are shown in the (**left**) panel.

The initial semi-quantitative assessment of the protective effect of heparinoids vis-à-vis FCS lysis by furin was carried out by monitoring the intensities of the proteolytic fragments of the FCS as a function of its exposure to furin in the presence of heparin oligomers; proteolysis in the heparinoid-free solution was used as the control experiment. Although both proteolytic fragments (YQTQTNSPRRAR and SVAS) were clearly visible in the LC/MS measurements, the signal generated by the former was notably higher, reflecting its polybasic character (Figure 5, left panel). Therefore, the semi-quantitative assessment of the efficiency of FCS processing by furin was carried out by measuring the absolute signal intensity of ions representing the larger proteolytic fragment. The kinetic plots of FCS proteolysis in deionized water (Figure 5, right panel) revealed a strong correlation between the heparinoid chain length and its effectiveness as a furin inhibitor (the weight concentrations of the three heparinoids used in these measurements were equal (100 μg), giving identical effective concentrations of the anionic monosaccharide residue units).

**Figure 6:**
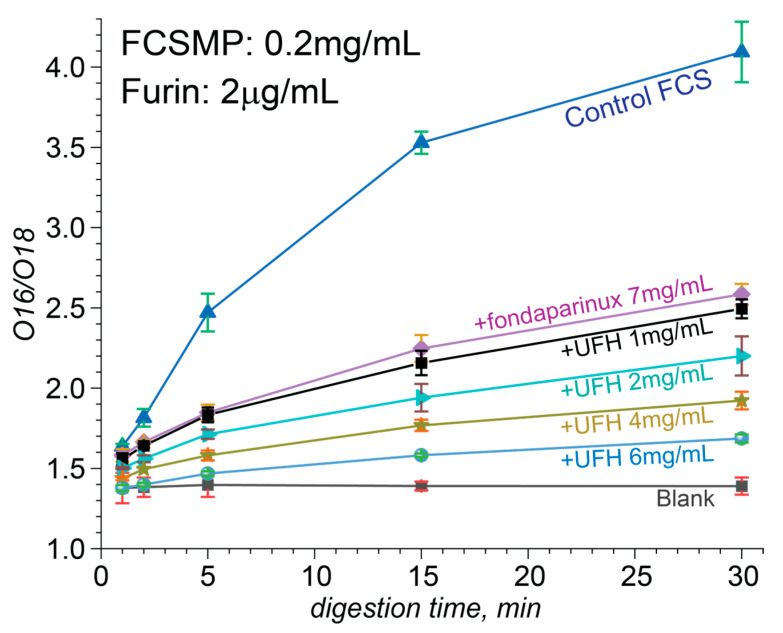
Quantitative studies of the proteolysis kinetics carried out using an O^18^-labeled internal standard at physiological ionic strength. O^18^/O^16^ peak ratio of proteolytic fragments at different unfractionated heparin concentrations ranging from 1 mg/mL to 6 mg/mL, plotted as a function of the digestion time (from 1 to 30 min).

The more precise quantitation of the proteolysis kinetics in the presence and absence of heparinoids was carried out using an O^18^-labeled internal standard (Figure S6), which was produced following the procedure introduced by Yao and Fenselau [36]. Consistent with the native MS results revealing a dramatic attenuation of the ability of short heparin oligomers to bind to the FCS at the physiological ionic strength (see Section 3.2), their protective effect vis-à-vis proteolysis was also strongly reduced by salt. Au contraire, the protective effect of UFH was much stronger and persisted even at the physiological ionic strength. The kinetic data presented in Figure 6 were acquired at the physiological ionic strength and used to evaluate the rates of FCS proteolytic processing at various heparin concentrations in solution. Based on these data, the IC_50_ value can be estimated as 0.6 ± 0.1 mg/mL (Figure S7).

## 4. Discussion

The therapeutic potential of heparin and related GAGs in areas as diverse as neurodegenerative diseases and bone tissue restoration has been widely acknowledged [37]; however, their clinical uses outside of the traditional role as anticoagulants remain disappointingly limited. The recent COVID-19 pandemic stimulated the use of heparin outside of its traditional therapeutic niche [38], but the early enthusiasm was somewhat curtailed by the concerns over the possible development of heparin-induced thrombocytopenia (HIT) [39] and its potential role in exacerbating the sequela of the coronavirus infection [40]. Historically, the risk of triggering HIT was mitigated by substituting heparin with shorter-chain antithrombin-activating GAGs, such as low-molecular-weight heparin, or synthetic heparin mimics [41]. In that respect, it is instructive to compare UFH to its shorter-chain derivatives and analogs vis-à-vis their ability to interfere with the proteolytic processing of the SARS-CoV-2 S glycoprotein furin cleavage site, the key step in the viral entry into human lung cells [13].

The polybasic furin cleavage site contains three arginine residues, and even the shortest heparin mimetic fondaparinux containing ten anionic groups (eight sulfates and two carboxylates) not only bound to the model peptide FCS efficiently under low-salt conditions, but in fact, was capable of bridging two such polycationic peptides (Figure 2). Switching from a chemically defined heparin analog to the relatively short heparin fragment dp6 allows the binding preferences of the polyanion to be investigated as a function of the total number of negative charges (from nine in (6,6,0) to twelve in (6,9,0)). Interestingly, the intensity distribution of ions representing the FCS·(6,*n*,0/1) complexes mirrors that of the (6,*n*,0/1) species in the absence of the peptide (Figure 3A,B), suggesting that even the lowest-charge heparin hexamers (6,6,0) and (6,6,1) are capable of binding to the single peptide in the low-salt solution. However, the intensity distribution of the polyanion-bridged peptide dimers FCS_2_·(6,*n*,0) shows a dramatic departure from this pattern, with only the highest-charge hexamers (6,8,0) and (6,9,0) lending themselves as effective peptide-bridging species (Figure 3C). This highlights the critical role played by the charges in FCS/dp6 interactions and points out that there is a certain minimum charge for the polyanions that allows them to form complexes with a specific stoichiometry. Indeed, the number of sulfate groups appears to trump the chain length for similarly-sized heparinoids as far as allowing a single polyanion to accommodate multiple peptides (e.g., the five-residue-long fondaparinux is capable of bridging two polybasic peptides, while the lowest-charge-density hexamer (6,6,0) lacks this capacity; see Table 1). For the heparin-derived oligomers, the average charge for the fixed-length species correlates with the chain length (an average of 2.7 sulfate groups per disaccharide unit [42]). Therefore, it is not surprising that the dp20 species can accommodate a significantly larger number of peptides, although this number also depends on the extent of the sulfation of a particular dp20 species (Table 1).

A further increase in the polyanion chain length while keeping the average charge density constant might be expected to have a mostly quantitative effect on the heparin association with the polybasic peptide, i.e., lead to a monotonic increase in the number of peptides accommodated by a single polyanion chain. Indeed, the highly charged dp20 species exhibited a four-fold increase in their FCS-binding capacity compared to the penta-saccharide fondaparinux, while the intact UFH molecules displayed an even larger peptide-binding capacity, consistent with the ten-fold increase in the average size over that of fondaparinux. However, there is also an important qualitative difference between UFH and its shorter-chain derivatives vis-à-vis FCS binding. The addition of even moderate amounts of salt (30 mM) to the solutions of shorter-chain heparinoids abrogated their ability to form stable complexes with the FCS, as reflected in native MS measurements (Figure S3), while the abundant ionic signal of UFH/FCS persisted at the physiological ionic strength (Figure 4). Furthermore, the level of inhibition of FCS proteolysis that can be achieved with UFH requires nearly an order of magnitude higher amount of the pentasaccharide fondaparinux (Figure 6).

**Figure 7:**
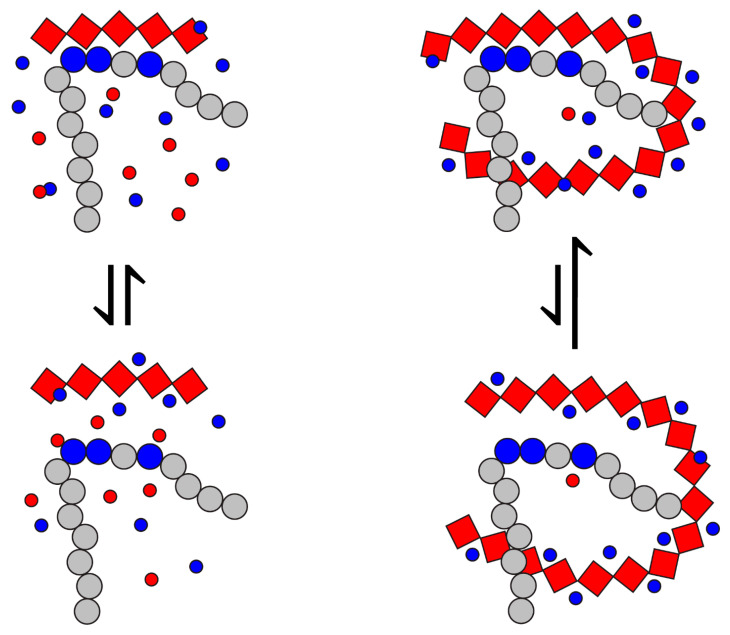
A schematic diagram of the effect of the polyanion chain length (red diamonds) on the local concentration of counter-anions balancing the positive charge of the polybasic peptide segment (large blue circles). The electrically neutral residues are depicted with gray circles; counterions are shown with small blue (cations) and red (anions) circles.

Clearly, despite their nearly identical chemical structure, UFH exhibits a significantly stronger ability to associate with the FCS under physiologically relevant conditions compared to its shorter-chain derivatives and analogs, which is also reflected in its much stronger potential as the peptide protector against the protease action. One factor that makes a long heparin chain distinct from that of shorter-chain oligomers and analogs is its reduced mobility (due to the significantly larger mass) and the presence of “excess” charges that participate in long-range interactions with the guanidinium groups of the three arginine residues within the furin cleavage site, despite not being in direct contact with them. This, in turn, is expected to decrease the number of small monovalent anions within the immediate vicinity of the FCS molecule (Figure 7). The free energy of the polyanion association with the polybasic peptide can be written as follows:

(1) $$∆G_{association}=∆G_{PC+PA}-\sum_{displ}∆G_{PC+CA}-\sum_{displ}∆G_{PA+CC},$$

where the first term reflects the gain due to the association of the two polyelectrolyte chains (an enthalpic gain due to maximizing the electrostatic attraction and an entropic loss due to the decrease in the conformational freedom within the binding segments), and the two other terms reflect the losses due to the displacement of the monovalent counterions from the binding interface. The extended heparin chain will provide additional contributions to the first term in Equation (1) due to the electrostatic attraction between the guanidinium groups of the FCS and the distal anionic saccharide residues of heparin. Kinetically, this is expected to result in a decrease in the *k*_OFF_ rate constant. Another difference between the long-chain heparin molecules and their shorter-chain fragments and analogs is the significant mass difference, which is reflected in a dramatic reduction in the mobility of the intact heparin molecules. This is expected to lead to the slower separation of the two chains (polycationic peptide and polyanionic GAG, *PC*, and *PA* in Equation (1)) away from each other following the transient dissociation of the complex. As a result, the slower diffusion-in of the monovalent counterions (especially the counter-anions, *CA* in Equation (1)) is expected, which should result in the less favorable contribution of the second term in Equation (1). Kinetically, this is expected to result in a notable increase in the *k*_ON_ rate constant. Therefore, the combination of the additional long-range electrostatic interactions stabilizing the complex and the impeded influx of the monovalent counterions during the transient dissociation events should lead to a dramatic increase in the stability of the polycation/polyanion complex in the case of intact heparin, as was observed experimentally.

One other important factor that was not explored in our work, but is also likely to give UFH an edge over the shorter-chain polyanions, is the avidity of its interaction with the furin cleavage sites within the context of the intact S glycoprotein. Prior simulations by Paiardi et al. demonstrated that heparin can bind along a path spanning the receptor-binding domain (RBD) to the S1/S2 furin cleavage site in the open conformation of the spike protein [43]. However, the potential of heparin to associate exclusively with (multiple) furin cleavage site(s) remains unexplored. Indeed, there are three identical furin cleavage sites within the S glycoprotein trimer, and the optimal protector against the proteolytic action of the cell surface serine proteases will be the one with the highest binding avidity (ideally, a single polyanion chain capable of binding simultaneously to all three furin cleavage sites within the stalk region of the trimeric S glycoprotein). The docking studies carried out with the ectodomain of the S glycoprotein (Figure S8) indicate that the minimal chain length required for bridging two furin cleavage sites within a single S glycoprotein trimer is 26, while 52 monosaccharide residues would enable the single polyanion chain to bind to all three sites. The size is approximately equal to the average chain length of UFH (*vide supra*), suggesting that all three furin cleavage sites within a trimeric spike can be covered by a single heparin chain, which would maximize the protection due to the high avidity of the interaction. Given that the distance between these sites is approximately 270 Å, which exceeds the persistence length of heparin (Kuhn segment length: ~9 nm [44]), the chain retains sufficient flexibility to accommodate multivalent binding.

Lastly, it should be mentioned that some mutations (such as D614G) can eliminate the need for the pre-fusion processing of the virus by cell surface proteases via enabling the endosomal uptake route [45]. Such mutants, of course, would be much less sensitive to heparin vis-à-vis the cell surface protease-mediated entry route. However, there is evidence that heparin can also inhibit the proteolytic activity of cathepsin L [46], the key enzyme controlling the endosomal route of viral entry into the host cell. Furthermore, due to its ability to activate antithrombin, heparin is directly involved in inhibiting coagulation factors (such as factor Xa), which has been shown to contribute to SARS-CoV-2 infectivity by processing the S glycoprotein furin cleavage site while in circulation, thereby enhancing viral entry [47]. This, as well as the previously demonstrated ability of heparin to interfere with the ACE2/S glycoprotein association [9,10], indicates that this century-old therapeutic can act as an effective and multi-prong antiviral agent, providing a rationale behind the apparent clinical success of this drug in the COVID-19 setting [48].

## 5. Conclusions

More than five years after the start of the COVID-19 pandemic, the SARS-CoV-2 virus maintains a substantial presence worldwide and continues to generate a significant number of new cases. Although the combination of herd immunity and an aggressive vaccination campaign have resulted in a dramatic decrease in COVID-19 related mortality, many other challenges remain unaddressed, such as the burden of long COVID [49]. Furthermore, COVID-19 contributes to mortality indirectly, placing previously hospitalized patients at an increased risk of death for various organ disorders for up to two and a half years following their discharge [50]. Therefore, there is still an urgent need to identify and optimize antiviral agents, especially those that are less sensitive (or indeed completely insensitive) to the rapid mutations of SARS-CoV-2. The ability of heparin to interfere with the proteolytic processing of the SARS-CoV-2 S glycoprotein demonstrated in this work highlights the antiviral potential of this century-old therapeutic. Furthermore, these anti-proteolytic properties of heparin may act synergistically with its previously reported ability to interfere with S-protein docking to the host cell surface receptor ACE2, giving rise to multi-modal antiviral protection. It is, therefore, not surprising that heparin’s ability to attenuate SARS-CoV-2 infectivity has been demonstrated in vitro [51], and its utility has been proven in the clinic as well [48].

Importantly, the apparent therapeutic effectiveness of heparin vis-à-vis coronavirus infections at the molecular level reflects a concerted action of multiple relatively non-specific long-range electrostatic interactions, rather than highly specific interactions arising due to a near-perfect surface complementarity at the binding interfaces. Such non-specific interactions have been mostly ignored within the reigning paradigm in medicinal chemistry. However, the critical roles played by cognate polyelectrolytes (e.g., GAGs and polyphosphates) in many biological processes indicate that the further growth of therapeutic strategies where non-specific electrostatic interactions play a central role is inevitable.

## Figures and Tables

**Table 1:** The highest number of polybasic peptides accommodated by a single fixed-length heparinoid.

		Chain Length (Number of Saccharide Residues)	Number of Negative Charges at pH 7/Number of Sulfate Groups	Max *n* (FCS Molecules Bound to a Single Chain
fondaparinux		5	10/8	2
dp6	(6,6,0)	6	9/6	1
	(6,7,0)	6	10/7	2 (low abundance)
	(6,7,1)	6	10/7	2
	(6,8,0)	6	11/8	2
	(6,8,1)	6	11/8	2
	(6,9,0)	6	12/9	2
dp20	(20,17,0)	20	27/17	8
	(20,24,n)	20	34/24	8
	(20,27,0)	20	37/27	8
	(20,28,1)	20	38/28	8

## Data Availability

The original data presented in the study are openly available in FigShare (doi 10.6084/m9.figshare.29144384).

## Supplementary Materials

The following supporting information can be downloaded at: https://www.mdpi.com/article/10.3390/biom15060778/s1, Table S1, Relative binding free energies of the top-ranked FCS–fondaparinux complexes; Figure S1: Molecular modeling of heparin dp6 and 2 FCS peptides; Figure S2: Full-range mass spectra of dp6 and the FCS/dp6 mixture; Figure S3: Mass spectra of the FCS/fondaparinux mixture at varying ionic strengths; Figure S4: Mass spectra of the FCS/dp20 mixtures; Figure S5: Deconvolution of the mass spectra of the FCS/dp20 mixtures; Figure S6: Isotopic distri-butions of the proteolytic fragment YQTQTNSPRRAR produced by O^18^ labeling and mixing the internal standard with the unlabeled fragment peptide; Figure S7: Inhibition of furin activity by UFH at physiological ionic strength as a function of heparin concentration; Figure S8: Docking of heparin dp30 with trimeric S glycoprotein ectodomain.

## References

[1] Carvajal J.J., García-Castillo V., Cuellar S.V., Campillay-Véliz C.P., Salazar-Ardiles C., Avellaneda A.M., Muñoz C.A., Retamal-Díaz A., Bueno S.M., González P.A. (2024). New insights into the pathogenesis of SARS-CoV-2 during and after the COVID-19 pandemic. Front. Immunol..

[2] Maison D.P., Tasissa H., Deitchman A., Peluso M.J., Deng Y., Miller F.D., Henrich T.J., Gerschenson M. (2025). COVID-19 clinical presentation, management, and epidemiology: A concise compendium. Front. Public Health.

[3] Vicenzi E., Canducci F., Pinna D., Mancini N., Carletti S., Lazzarin A., Bordignon C., Poli G., Clementi M. (2004). Coronaviridae and SARS-associated coronavirus strain HSR1. Emerg. Infect. Dis..

[4] Liu J., Li J., Arnold K., Pawlinski R., Key N.S. (2020). Using heparin molecules to manage COVID-2019. Res. Pract. Thromb. Haemost..

[5] Hippensteel J.A., LaRiviere W.B., Colbert J.F., Langouët-Astrié C.J., Schmidt E.P. (2020). Heparin as a Therapy for COVID-19: Current Evidence and Future Possibilities. Am. J. Physiol. Lung. Cell. Mol. Physiol..

[6] Miesbach W., Makris M. (2020). COVID-19: Coagulopathy, Risk of Thrombosis, and the Rationale for Anticoagulation. Clin. Appl. Thromb. Hemost..

[7] Lindahl U., Li J.P. (2020). Heparin—An old drug with multiple potential targets in COVID-19 therapy. J. Thromb. Haemost..

[8] Lawler P.R., Goligher E.C., Berger J.S., Neal M.D., McVerry B.J., Nicolau J.C., Gong M.N., Carrier M., Rosenson R.S., Reynolds H.R. (2021). Therapeutic Anticoagulation with Heparin in Noncritically Ill Patients with COVID-19. N. Engl. J. Med..

[9] Mycroft-West C.J., Su D., Pagani I., Rudd T.R., Elli S., Gandhi N.S., Guimond S.E., Miller G.J., Meneghetti M.C.Z., Nader H.B. (2020). Heparin Inhibits Cellular Invasion by SARS-CoV-2: Structural Dependence of the Interaction of the Spike S1 Receptor-Binding Domain with Heparin. Thromb. Haemost..

[10] Yang Y., Du Y., Kaltashov I.A. (2020). The utility of native MS for understanding the mechanism of action of repurposed therapeutics in COVID-19: Heparin as a disruptor of the SARS-CoV-2 interaction with its host cell receptor. Anal. Chem..

[11] Yang Y., Ivanov D.G., Kaltashov I.A. (2021). The challenge of structural heterogeneity in the native mass spectrometry studies of the SARS-CoV-2 spike protein interactions with its host cell-surface receptor. Anal. Bioanal. Chem..

[12] Kim S.Y., Jin W., Sood A., Montgomery D.W., Grant O.C., Fuster M.M., Fu L., Dordick J.S., Woods R.J., Zhang F. (2020). Characterization of heparin and severe acute respiratory syndrome-related coronavirus 2 (SARS-CoV-2) spike glycoprotein binding interactions. Antivir. Res..

[13] Hoffmann M., Kleine-Weber H., Pöhlmann S. (2020). A Multibasic Cleavage Site in the Spike Protein of SARS-CoV-2 Is Essential for Infection of Human Lung Cells. Mol. Cell.

[14] Cardin A.D., Weintraub H.J. (1989). Molecular modeling of protein-glycosaminoglycan interactions. Arteriosclerosis.

[15] Carabelli A.M., Peacock T.P., Thorne L.G., Harvey W.T., Hughes J., Peacock S.J., Barclay W.S., de Silva T.I., Towers G.J., Robertson D.L. (2023). SARS-CoV-2 variant biology: Immune escape, transmission and fitness. Nat. Rev. Microbiol..

[16] Barile E., Baggio C., Gambini L., Shiryaev S.A., Strongin A.Y., Pellecchia M. (2020). Potential Therapeutic Targeting of Coronavirus Spike Glycoprotein Priming. Molecules.

[17] Ivachtchenko A.V., Khvat A.V., Shkil D.O. (2024). Development and Prospects of Furin Inhibitors for Therapeutic Applications. Int. J. Mol. Sci..

[18] Seidah N.G., Prat A. (2012). The biology and therapeutic targeting of the proprotein convertases. Nat. Rev. Drug Discov..

[19] Johnson B.A., Xie X., Bailey A.L., Kalveram B., Lokugamage K.G., Muruato A., Zou J., Zhang X., Juelich T., Smith J.K. (2021). Loss of furin cleavage site attenuates SARS-CoV-2 pathogenesis. Nature.

[20] Sasaki M., Toba S., Itakura Y., Chambaro H.M., Kishimoto M., Tabata K., Intaruck K., Uemura K., Sanaki T., Sato A. (2021). SARS-CoV-2 Bearing a Mutation at the S1/S2 Cleavage Site Exhibits Attenuated Virulence and Confers Protective Immunity. mBio.

[21] Willett B.J., Grove J., MacLean O.A., Wilkie C., De Lorenzo G., Furnon W., Cantoni D., Scott S., Logan N., Ashraf S. (2022). SARS-CoV-2 Omicron is an immune escape variant with an altered cell entry pathway. Nat. Microbiol..

[22] Molloy S.S., Bresnahan P.A., Leppla S.H., Klimpel K.R., Thomas G. (1992). Human furin is a calcium-dependent serine endoprotease that recognizes the sequence Arg-X-X-Arg and efficiently cleaves anthrax toxin protective antigen. J. Biol. Chem..

[23] Friesner R.A., Banks J.L., Murphy R.B., Halgren T.A., Klicic J.J., Mainz D.T., Repasky M.P., Knoll E.H., Shelley M., Perry J.K. (2004). Glide: A new approach for rapid, accurate docking and scoring. 1. Method and assessment of docking accuracy. J. Med. Chem..

[24] Roos K., Wu C., Damm W., Reboul M., Stevenson J.M., Lu C., Dahlgren M.K., Mondal S., Chen W., Wang L. (2019). OPLS3e: Extending Force Field Coverage for Drug-Like Small Molecules. J. Chem. Theory. Comput..

[25] Obradovic Z., Peng K., Vucetic S., Radivojac P., Brown C.J., Dunker A.K. (2003). Predicting intrinsic disorder from amino acid sequence. Proteins.

[26] Walls A.C., Park Y.J., Tortorici M.A., Wall A., McGuire A.T., Veesler D. (2020). Structure, Function, and Antigenicity of the SARS-CoV-2 Spike Glycoprotein. Cell.

[27] Wrapp D., Wang N., Corbett K.S., Goldsmith J.A., Hsieh C.L., Abiona O., Graham B.S., McLellan J.S. (2020). Cryo-EM structure of the 2019-nCoV spike in the prefusion conformation. Science.

[28] Cai Y., Zhang J., Xiao T., Peng H., Sterling S.M., Walsh R.M., Rawson S., Rits-Volloch S., Chen B. (2020). Distinct conformational states of SARS-CoV-2 spike protein. Science.

[29] Petitou M., van Boeckel C.A.A. (2004). A synthetic antithrombin III binding pentasaccharide is now a drug! What comes next?. Angew. Chem. Int. Ed. Engl..

[30] Kayitmazer A.B., Quinn B., Kimura K., Ryan G.L., Tate A.J., Pink D.A., Dubin P.L. (2010). Protein Specificity of Charged Sequences in Polyanions and Heparins. Biomacromolecules.

[31] Niu C., Zhao Y., Bobst C.E., Savinov S.N., Kaltashov I.A. (2020). Identification of Protein Recognition Elements within Heparin Chains Using Enzymatic Foot-Printing in Solution and Online SEC/MS. Anal. Chem..

[32] Henriksen J., Ringborg L.H., Roepstorrf P. (2004). On-line size-exclusion chromatography/mass spectrometry of low molecular mass heparin. J. Mass Spectrom..

[33] Abzalimov R.R., Kaltashov I.A. (2010). Electrospray ionization mass spectrometry of highly heterogeneous protein systems: Protein ion charge state assignment via incomplete charge reduction. Anal. Chem..

[34] Zhao Y., Abzalimov R.R., Kaltashov I.A. (2016). Interactions of Intact Unfractionated Heparin with Its Client Proteins Can Be Probed Directly Using Native Electrospray Ionization Mass Spectrometry. Anal. Chem..

[35] Yang Y., Du Y., Ivanov D., Niu C., Clare R., Smith J.W., Nazy I., Kaltashov I.A. (2024). Molecular architecture and platelet-activating properties of small immune complexes assembled on heparin and platelet factor 4. Commun. Biol..

[36] Fenselau C., Yao X. (2009). ^18^O_2_-Labeling in quantitative proteomic strategies: A status report. J. Proteome Res..

[37] Hogwood J., Mulloy B., Lever R., Gray E., Page C.P. (2023). Pharmacology of Heparin and Related Drugs: An Update. Pharmacol. Rev..

[38] Tritschler T., Le Gal G., Brosnahan S., Carrier M. (2022). POINT: Should Therapeutic Heparin Be Administered to Acutely Ill Hospitalized Patients with COVID-19? Yes. Chest.

[39] Sugraliyev A.B. (2024). Heparin-Induced Thrombocytopenia. Kardiologiia.

[40] Favaloro E.J., Henry B.M., Lippi G. (2021). The complicated relationships of heparin-induced thrombocytopenia and platelet factor 4 antibodies with COVID-19. Int. J. Lab. Hematol..

[41] Greinacher A. (2015). CLINICAL PRACTICE. Heparin-Induced Thrombocytopenia. N. Engl. J. Med..

[42] Toida T., Yoshida H., Toyoda H., Koshiishi I., Imanari T., Hileman R.E., Fromm J.R., Linhardt R.J. (1997). Structural differences and the presence of unsubstituted amino groups in heparan sulphates from different tissues and species. Biochem. J..

[43] Paiardi G., Richter S., Oreste P., Urbinati C., Rusnati M., Wade R.C. (2022). The binding of heparin to spike glycoprotein inhibits SARS-CoV-2 infection by three mechanisms. J. Biol. Chem..

[44] Pavlov G., Finet S., Tatarenko K., Korneeva E., Ebel C. (2003). Conformation of heparin studied with macromolecular hydrodynamic methods and X-ray scattering. Eur. Biophys. J..

[45] Guo C., Tsai S.J., Ai Y., Li M., Anaya E., Pekosz A., Cox A., Gould S.J. (2022). The D614G mutation redirects SARS-CoV-2 spike to lysosomes and suppresses deleterious traits of the furin cleavage site insertion mutation. Sci. Adv..

[46] Higgins W.J., Fox D.M., Kowalski P.S., Nielsen J.E., Worrall D.M. (2010). Heparin enhances serpin inhibition of the cysteine protease cathepsin L. J. Biol. Chem..

[47] Kastenhuber E.R., Mercadante M., Nilsson-Payant B., Johnson J.L., Jaimes J.A., Muecksch F., Weisblum Y., Bram Y., Chandar V., Whittaker G.R. (2022). Coagulation factors directly cleave SARS-CoV-2 spike and enhance viral entry. eLife.

[48] Spyropoulos A.C., Goldin M., Giannis D., Diab W., Wang J., Khanijo S., Mignatti A., Gianos E., Cohen M., Sharifova G. (2021). Efficacy and Safety of Therapeutic-Dose Heparin vs Standard Prophylactic or Intermediate-Dose Heparins for Thromboprophylaxis in High-risk Hospitalized Patients with COVID-19: The HEP-COVID Randomized Clinical Trial. JAMA Intern. Med..

[49] Sachs J.D., Karim S.S.A., Aknin L., Allen J., Brosbøl K., Colombo F., Barron G.C., Espinosa M.F., Gaspar V., Gaviria A. (2022). The Lancet Commission on lessons for the future from the COVID-19 pandemic. Lancet.

[50] Tubiana S., Rontani M., Herlemont P., Dray-Spira R., Zureik M., Weill A., Duval X., Burdet C. (2025). Long-term health outcomes following hospitalisation for COVID-19: A 30- month cohort analysis. Infect. Dis..

[51] Lee L.Y.Y., Suryadinata R., McCafferty C., Ignjatovic V., Purcell D.F.J., Robinson P., Morton C.J., Parker M.W., Anderson G.P., Monagle P. (2022). Heparin Inhibits SARS-CoV-2 Replication in Human Nasal Epithelial Cells. Viruses.

