# Supplementary material for: The Role of Long-Range Non-Specific Electrostatic Interactions in Inhibiting the Pre-Fusion Proteolytic Processing of the SARS-CoV-2 S Glycoprotein by Heparin
Source: Biomolecules. 2025 May 28;15(6):778. doi: 10.3390/biom15060778 (PMC12191132; doi:10.3390/biom15060778)

Supporting Information for

The role of long-range non-specific electrostatic interactions in inhibiting the pre-fusion proteolytic processing of the SARS-CoV-2 S glycoprotein by heparin

Yi Du, Yang Yang, Son N. Nguyen and Igor A. Kaltashov

Department of Chemistry, University of Massachusetts-Amherst, Amherst, MA 01003, USA

Table of Contents

Supplementary method

Table S1. Relative binding free energies (ΔG, kcal/mol) of top-ranked FCS–fondaparinux complexes calculated via Prime MM–GBSA refinement of Glide XP-docked poses.

Figure S1. Molecular modeling of heparin dp6 and 2 FCS peptides.

Figure S2. Full-range mass spectra of an aqueous solutions of dp6 (0.052 mg/mL) and the FCS/dp6 mixture (0.051 mg/mL and 0.052 mg/mL, respectively) in deionized water.

Figure S3. Mass spectra of aqueous solutions of the FCS/fondaparinux mixture (0.011 and 0.010mg/ml, respectively) in varies ionic strengths.

Figure S4. Mass spectra of aqueous solutions of the FCS/dp20 mixtures (0.027-1.09 mg/mL and 0.174 mg/mL, respectively).

Figure S5. Deconvolution of the mass spectra of the FCS/dp20 mixtures.

Figure S6. Isotopic distributions of the +2 charge state of the proteolytic fragment YQTQTNSPRRAR produced by O^{18} labeling (internal standard, black trace) and mixing the internal standard with the unlabeled fragment peptide generated by 30-min furin digestion of FCS (red trace).

Figure S7. Inhibition of furin activity by UFH vis-à-vis FCS processing at physiological ionic strength as a function of heparin concentration in solution.

Figure S8. Docking of heparin dp30 with trimeric S glycoprotein ectodomain

Supplementary method

Molecular dynamics: The initial structure of spike protein was taken from the crystal structure (PDB: **6VXX**). The missing furin cleavage site was added using Prime application then energy minimized. The Schrödinger Protein Preparation Wizard tool was used to optimize the proteins by removing water molecules and adding the missing hydrogen atoms, side chains, and bond orders. The fully sulfated heparin chains were generated by modifying the solution structure of dp24 (PDB: **3IRJ**) to incorporate the appropriate modifications. Subsequently, the chains were either shortened or extended to achieve the desired length. The model peptide was constructed from the known sequence YQTQTNSPRRARSVAS and energy minimized then run an additional 3ns simulation for further minimization. All SPC water systems were built by system builder as a Desmond application in Schrödinger Suite. In all cases, the periodic boxes employed in this study were orthorhombic in shape, with a buffer region of 10 Å maintained in all three dimensions between the solute and the boundaries of the box. This buffer helps to minimize interactions between the solute and its periodic images, ensuring accurate simulations and reducing boundary effects. The proper amount of Na+ ion and Cl- ion were added to neutralize the system and, for some studies, reach the salt concentration corresponding to the physiological ionic strength (150 mM). The solvated FCS/heparin systems were first equilibrated using two rounds of NVT simulations, which would be slowly heated up from 10 K to 310 K in a total of 100 ps and 12 ps, respectively, during which all protein heavy atoms were restrained using harmonic potentials. Another two rounds of 12-ps NPT simulations, where protein heavy atoms were similarly restrained, were executed at 1 atm, 10 K and 310 K, respectively. The next NPT simulation was then carried out at 310 K and 1 atm for a total of 24 ps with no restraints on protein heavy atoms for pre-equilibration. The production simulations were run under NPT conditions at 310 K and 1 atm for various time lengths. Recording interval is set as 100 ps. Noose-Hoover thermostat was used to control the temperature and barostat with volume move attempt every 2 ps to control the pressure. Lengths of all bonds involving hydrogen atoms were constrained using the SHAKE algorithm to allow for an integration time step of 2 fs with RESPA algorithm. Long-range electrostatic interactions were treated using the particle mesh Ewald method, and the short-range van der Waals interactions were treated with the cutoff at 9 Å. No enhanced sampling methods are needed as the complex formations are convergent at the brute-force MD simulation timescale.

Table S1. Relative binding free energies (ΔG, kcal/mol) of top-ranked FCS–fondaparinux and FCS-heparin dp6 complexes calculated via Prime MM–GBSA refinement of Glide XP-docked poses, where MMGBSA dG Bind = Complex – Receptor –Ligand and MMGBSA dG Bind(NS) = Complex – Receptor(from optimized complex) –Ligand(from optimized complex) = MMGBSA dG Bind – Receptor Strain – Ligand Strain. NS in the table is no strain; it is the binding energy without considering for the receptor and ligand conformational changes needed for the formation of complex.

Docking of FCS and fondaparinux	MMGBSA dG Bind	MMGBSA dG Bind Coulomb	MMGBSA dG Bind(NS)	MMGBSA dG Bind(NS) Coulomb
Fondaparinux Pose1	-48.13	-311.77	-64.38	-310.69
Fondaparinux Pose2	-50.8	-268.21	-62.25	-274.96
Fondaparinux Pose3	-38.03	-255.62	-65.76	-278.96
Fondaparinux Pose4	-40.88	-285.22	-62.06	-298.94
Fondaparinux Pose5	-38.55	-230	-52	-245.81
Fondaparinux Pose6	-48.14	-288.71	-61.23	-281.49
dp6 pose1	-51.03	-341.32	-67.32	-347.64
dp6 pose2	-45.21	-301.76	-57.3	-289.25
dp6 pose3	-36.72	-355.46	-53.54	-348.27
dp6 pose4	-42.15	-337.91	-60.12	-337.81
dp6 pose5	-44.25	-339.48	-57.09	-324.04
FCS/(6,9,0)	-32.07	-429.70	-37.27	-423.82

Figure S1. Molecular modeling of heparin dp6 and 2 FCS peptides. The gyration radius collapse within the initial 100ns and 50ns in 150mM NaCl (black) and pure water (red), respectively. The snapshots shows the evolution of the extended structure towards the polyanion-bridged peptide dimer under physiological ionic strength.

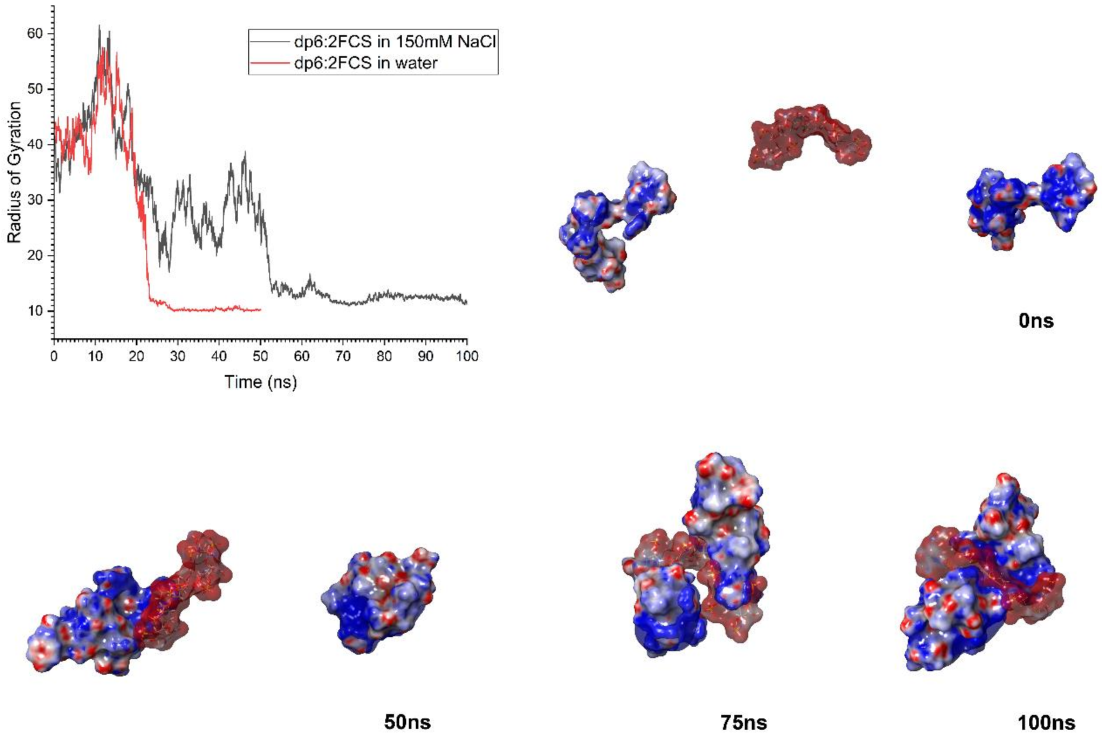

Figure S2. Full-range mass spectra of an aqueous solutions of dp6 (0.052 mg/mL) and the FCS/dp6 mixture (0.051 mg/mL and 0.052 mg/mL, respectively). The numbers in parentheses indicate the chain length, the number of sulfate groups and the number of acetyl groups within the chain. Complexes corresponding to dp6 bound to one or two FCS peptides are observed.

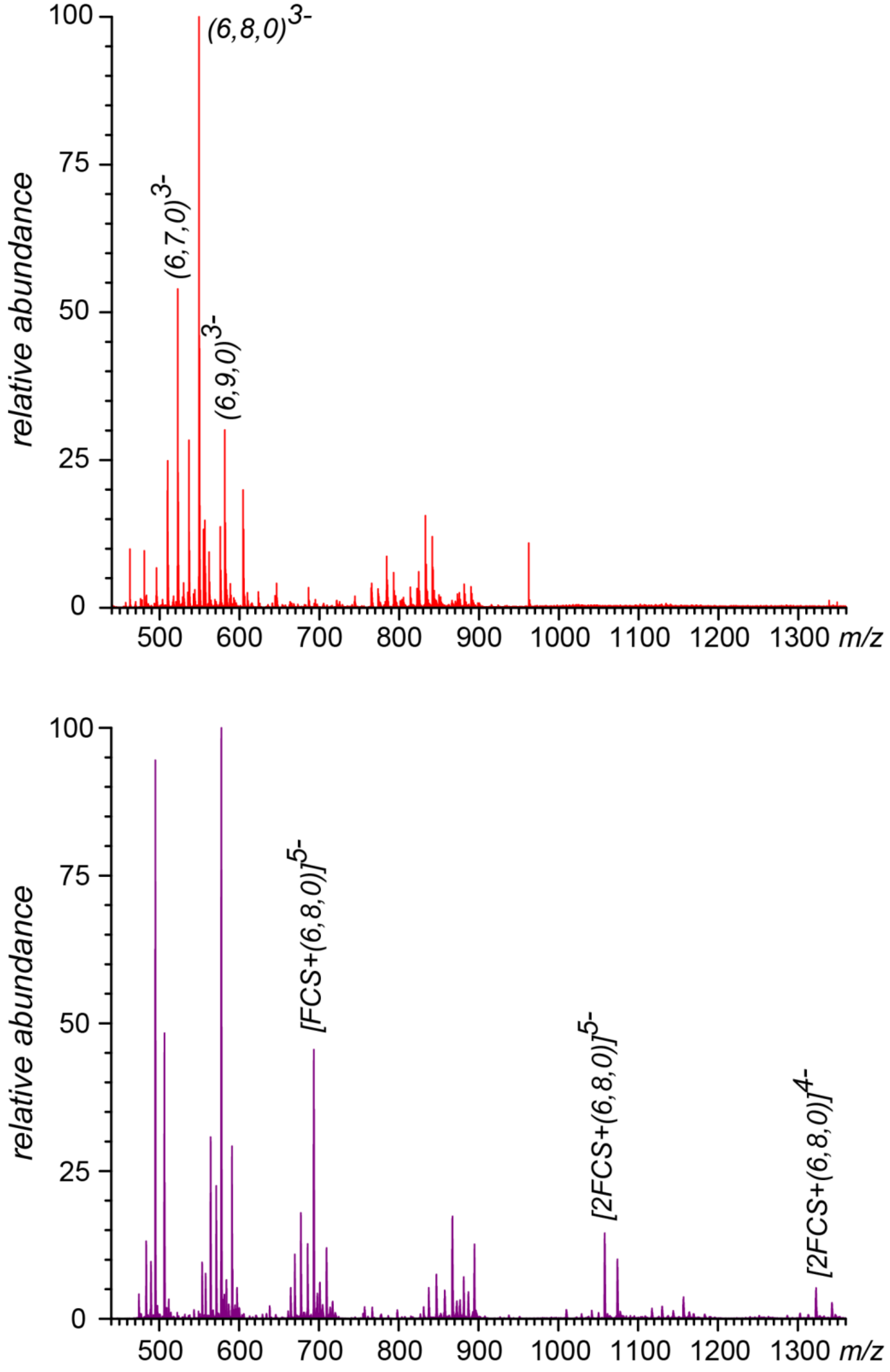

Figure S3. Mass spectra of aqueous solutions of fondaparinux alone (29 µM, black) and in complex with an equimolar amount of FCS are shown under various buffer conditions: deionized water (red), 10 mM ammonium acetate (blue), 25 mM ammonium acetate (green), and 150 mM ammonium acetate (purple). A set of zoomed-in spectra is provided to highlight key features, while the full spectra are displayed in the top right corner. Notably, the fondaparinux/FCS complex signal is dramatically reduced at 10 mM ammonium acetate and is completely absent at 25 mM, indicating high sensitivity of the complex to ionic strength.

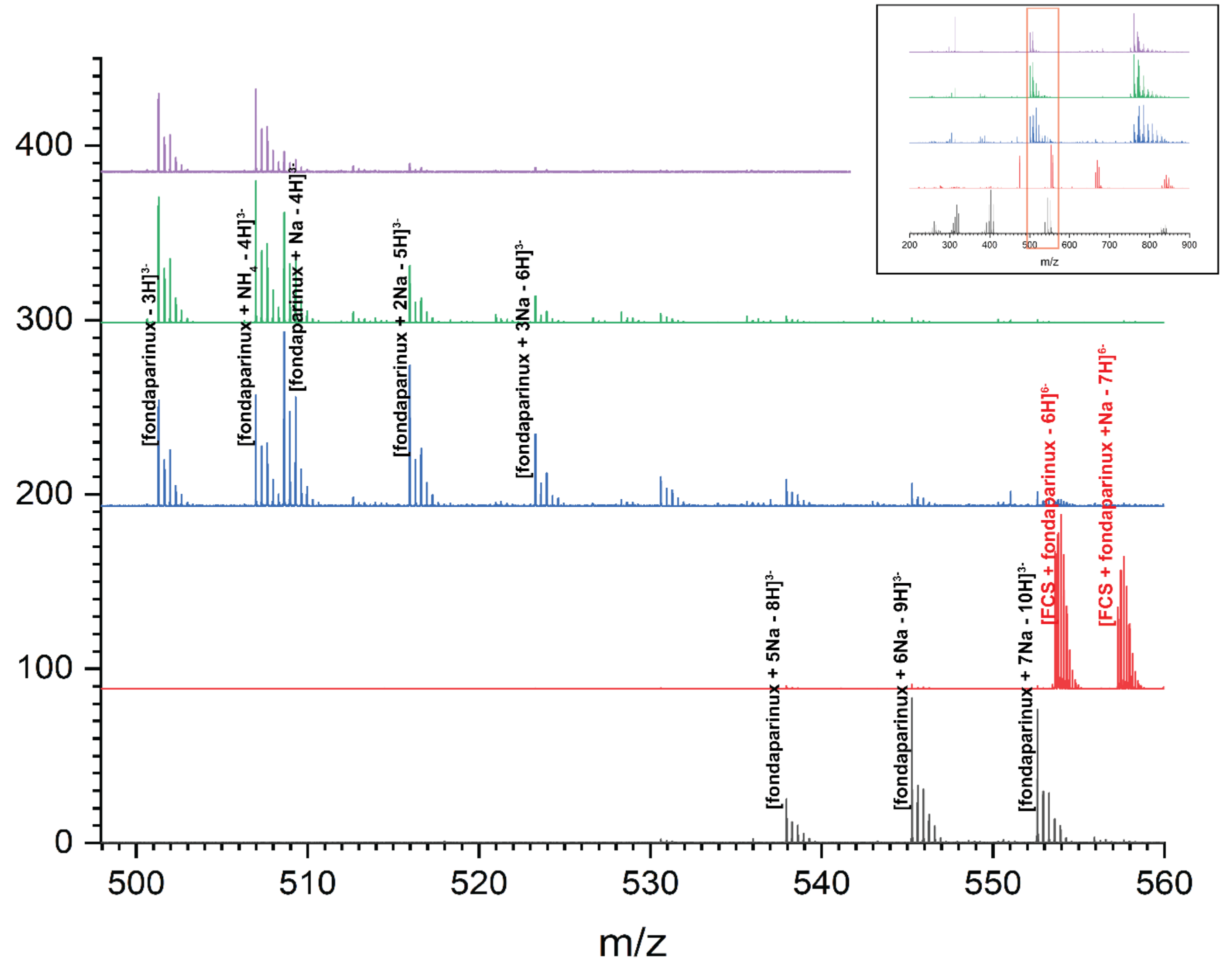

Figure S4. Mass spectra of aqueous solutions of dp20/FCS mixtures, with FCS at a fixed concentration of 0.174 mg/mL and dp20 ranging from 0.027 to 1.09 mg/mL, are shown. The accompanying table summarizes the average masses derived from the spectra, along with the estimated number of FCS molecules bound per dp20 chain. A deconvoluted spectrum of the 1:10 dp20/FCS mixture is presented in Fig. S5.

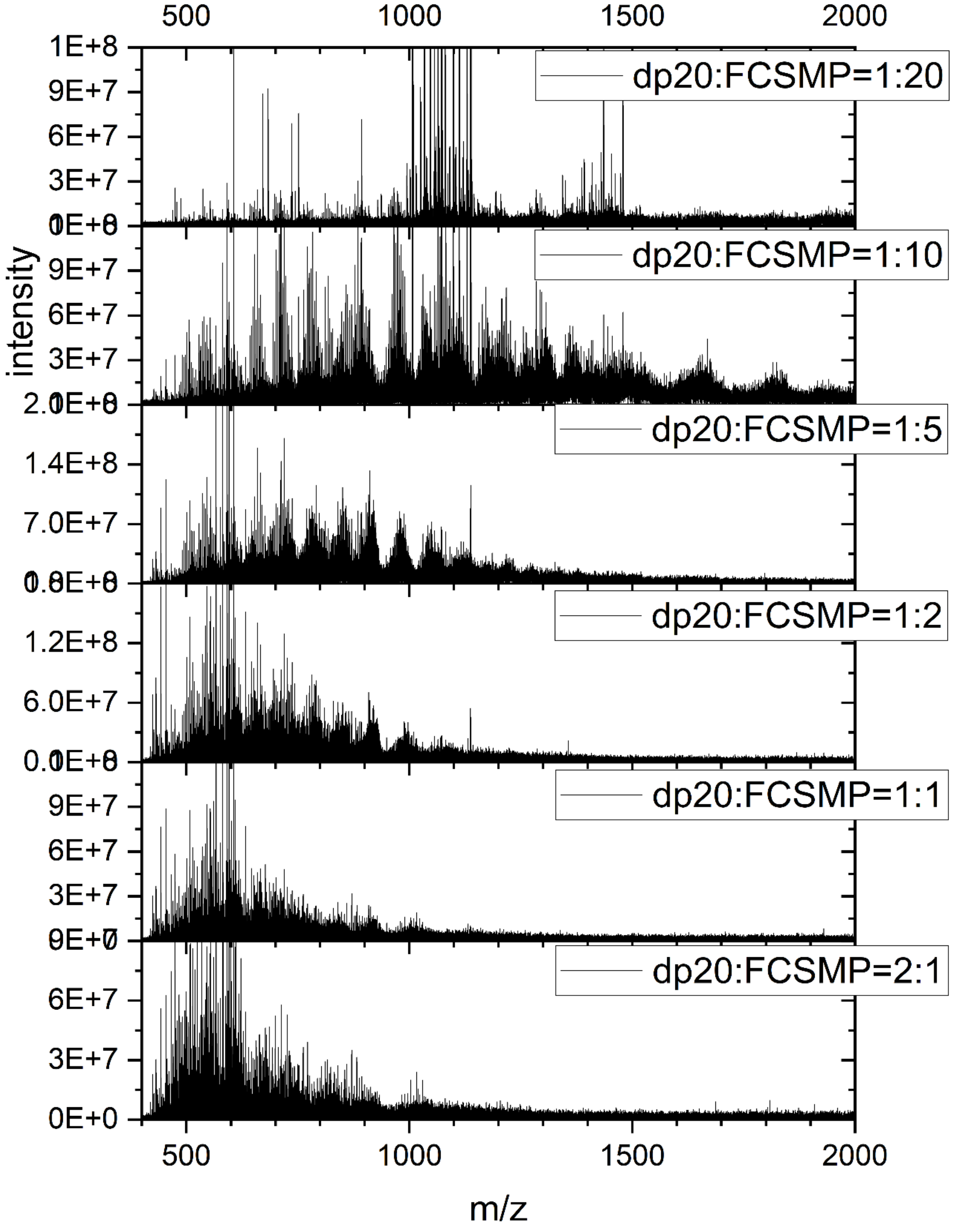

Heparin dp20:FCSMP molar ratio	Average Mass	Calculated n (FCS bound to a single chain)
2:1	~7282 Da	1
1:1	~7282 Da	1
1:2	~8705 Da	2
	~10881 Da	3
	~12060 Da	4
1:5	~7318 Da	1
	~9233 Da	2
	~10881 Da	3
	~13164 Da	4
	~14601 Da	5
	~16808 Da	6
1:10	~7318 Da	1
	~9151 Da	2
	~10643 Da	3
	~12562 Da	4
	~14565 Da	5
	~16426 Da	6
	~18282 Da	7
	~19319 Da	8

Figure S5. Although the masses were initially calculated manually (Panel **A**), the deconvoluted mass spectrum of the dp20/FCS 1:10 mixture (1.09 mg/mL dp20 and 0.174 mg/mL FCS) was subsequently obtained using UniDec (Panel **B**). Several regions of the spectrum could be interpreted based on the mass differences between adjacent peaks, consistent with varying degrees of sulfation and acetylation. Representative mass ranges—7500–8300 Da and 9600–10,100 Da—correspond to dp20/FCS complexes with stoichiometries of 1:1 and 1:2, respectively (Panels **C** and **D**).

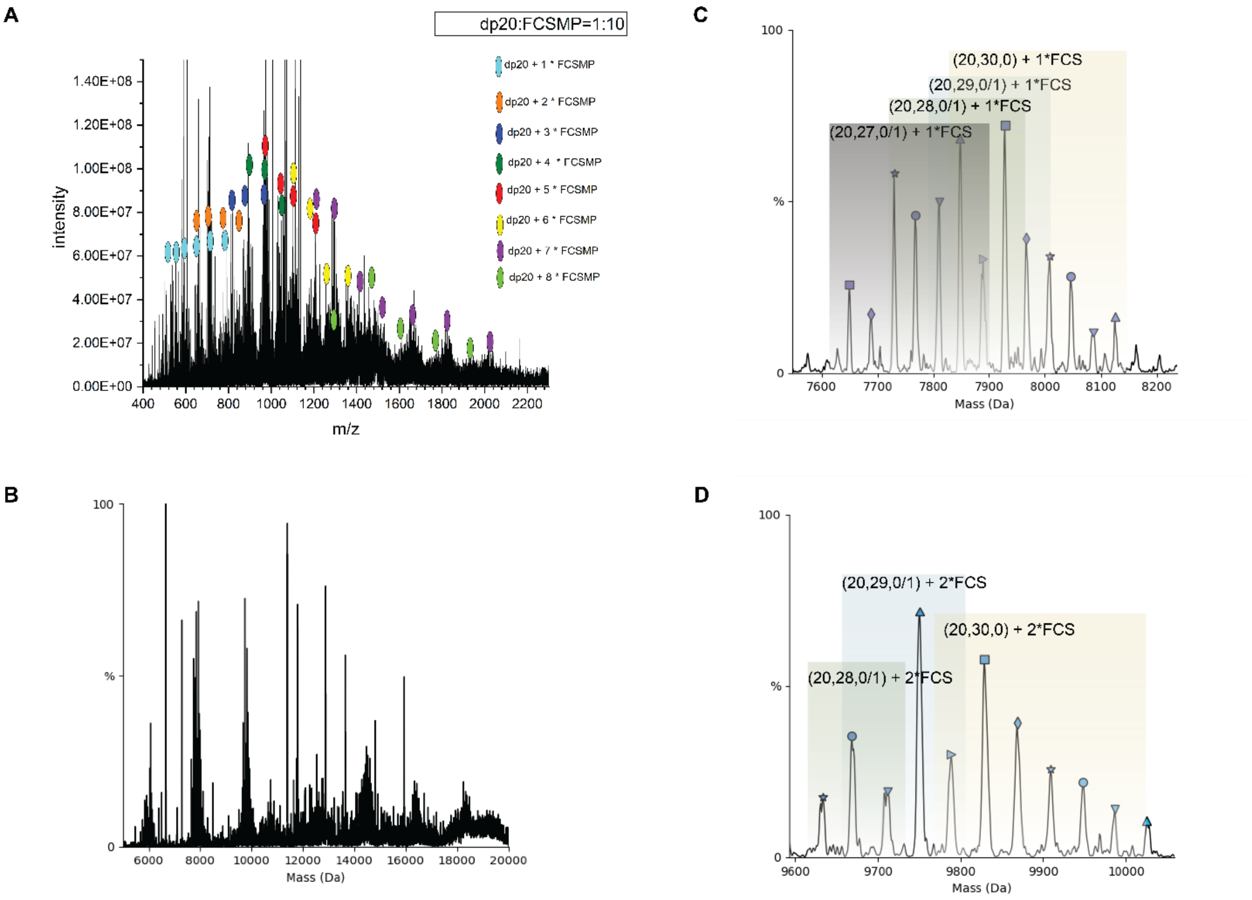

Figure S6. Isotopic distributions of the +2 charge state of the proteolytic fragment YQTQTNSPRRAR produced by O^{18} labeling (internal standard, black trace) and mixing the internal standard with the unlabeled fragment peptide generated by 30-min furin digestion of FCS (red trace).

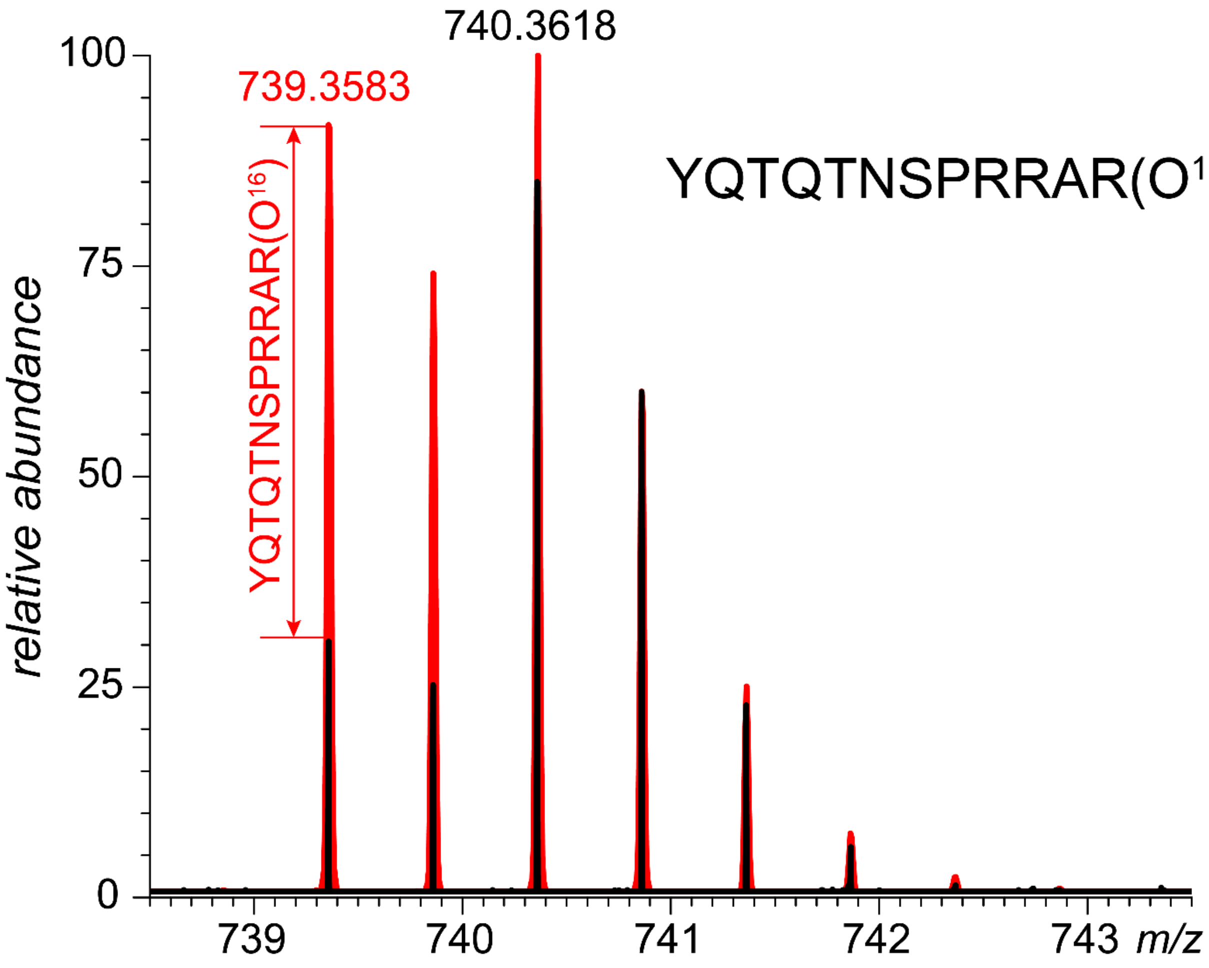

Figure S7. Inhibition of furin activity by UFH vis-à-vis FCS processing at physiological ionic strength as a function of heparin concentration in solution. IC_{50} value is estimated as 0.6±0.1 mg/mL.

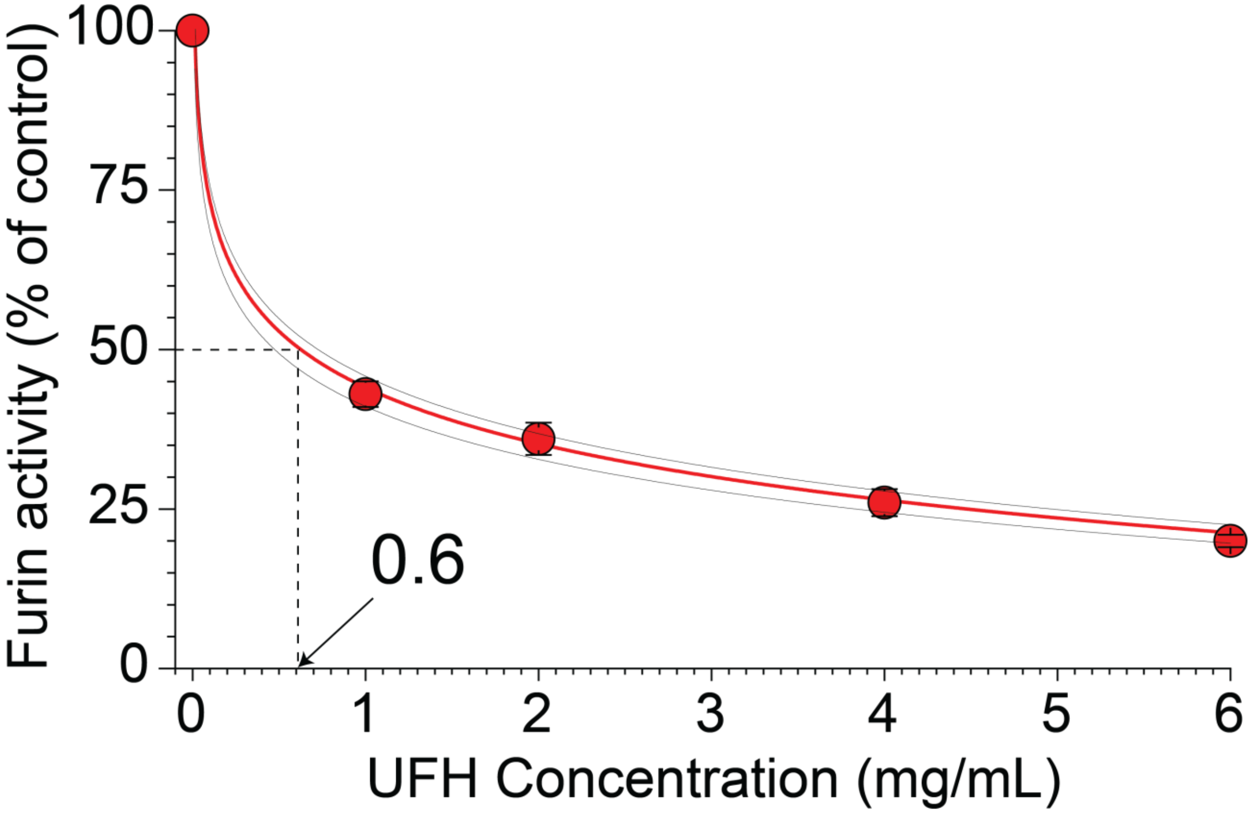

Figure S8. Docking of a long heparin chain (dp30) with trimeric S glycoprotein ectodomain. Shown here is an illustrative comparison of the spatial dimensions of dp30 relative to the S protein, highlighting the difference between the length of heparin dp30 and the distance of two furin cleavage sites.

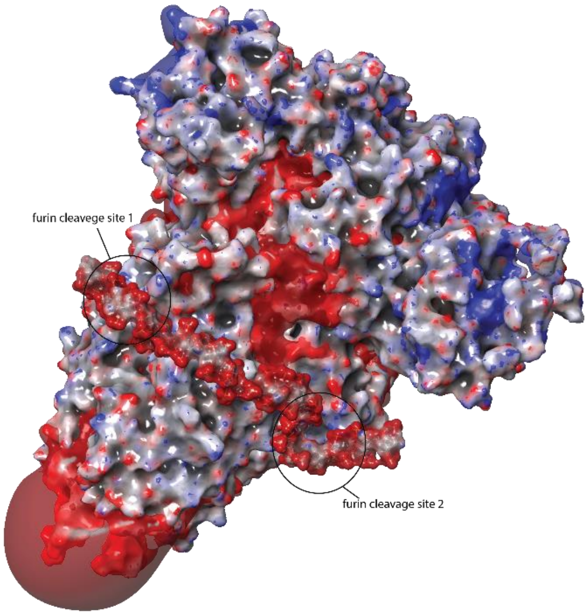

Supplement: Supplementary file 1 [file biomolecules-15-00778-s001.zip › biomolecules-3590492-supplementary.pdf]
